# Infrastructuring European scientific integration: Heterogeneous meanings of the European biobanking infrastructure BBMRI–ERIC

**DOI:** 10.1177/03063127231162629

**Published:** 2023-06-12

**Authors:** Erik Aarden

**Affiliations:** University of Klagenfurt, Klagenfurt, Austria

**Keywords:** Europe, biobanking, research infrastructures, research policy, European integration, infrastructural inversion

## Abstract

While transnational research infrastructure projects long preceded the formal integration process that created the European Union, their advancement is an increasingly central part of EU research policy and of European integration in general. This paper analyses the Biobanking and Biomolecular Resources Research Infrastructure–European Research Infrastructure Consortium (BBMRI–ERIC) as a recent example of institutionalized scientific collaboration in Europe that has formally been established as part of EU science policy. BBMRI–ERIC, a network of European biobanks, is expected to contribute to both European science and European integration. Yet its achievements in these domains are interpreted differently by various actors involved. This paper draws on STS conceptualizations of infrastructures as relational, experimental, and promissory assemblages. These support the formulation of a working definition of research infrastructures that in turn helps to explore the heterogeneous meanings attributed to BBMRI–ERIC. The paper describes the creation of this distributed European research infrastructure, and divergent understandings of what it means for BBMRI–ERIC to be *distributed*, to be *European* and to be a *research infrastructure*. This analysis demonstrates how building a research infrastructure is also an effort to define what it means to be European—a process in which what is European about science and what science can do for Europe is continuously (re-)imagined, contested and negotiated.

The history of European integration has long involved the establishment of transnational infrastructures of science, technology and expertise (Kaiser & Schot, 2014; Schipper & Schot, 2011). In the second half of the twentieth century this has included the creation of European research infrastructures, which were built on the hope of stimulating cross-border collaboration in the aftermath of World War II. The nuclear research organization CERN is perhaps the best-known example of such an effort to simultaneously build a shared research programme and a sense of being ‘European’ (Mobach & Felt, 2022).

Such research efforts long developed independently from and in parallel to the formal economic and political proceedings that created the European Union (Papon, 2004), to form an example of what Misa and Schot (2005) have termed the ‘hidden’ integration of Europe. Only more recently have scientific collaboration and its institutionalization in research infrastructures become part of the ‘formal’ integration process of the European Union. Activities such as the development of a ‘roadmap’ of prioritized European research infrastructures by the European Strategy Forum for Research Infrastructure (ESFRI), or the development of a dedicated legal framework—the European Research Infrastructure Consortium (ERIC)—by the European Commission illustrate the increasing prominence of research infrastructures, Nor is the European Union the only political unit developing ‘roadmaps’ for research infrastructures: in 2016, both South Africa and Australia released research infrastructure roadmaps of their own (Department of Education, 2022; Department of Science and Technology, 2016).

This paper analyses the Biobanking and Biomolecular Resources Research Infrastructure–European Research Infrastructure Consortium (BBMRI–ERIC) as an example of a recent cohort of institutionalized scientific collaborations in Europe that have formally been established within the confines of EU science policy. The biobanking infrastructure BBMRI–ERIC is of particular interest as one of the earliest and allegedly largest infrastructures governed according to the European Research Infrastructure Consortium (ERIC) legal framework. Moreover, it corresponds with ongoing investments in biomedical research that both aim to support science and industry and advance European integration and identity (Aarden et al., 2021; Marelli & Testa, 2017). In the next section, I expand on BBMRI–ERIC’s relation to the historical development of European research infrastructures, as well as its establishment and organization. I then turn to the question how to conceptualize European research infrastructures, which largely remain ill-defined to date. I propose a working definition that I subsequently employ to explore different meanings attributed to BBMRI–ERIC as a *distributed European research infrastructure*. I explore the heterogeneous relations between scientific and European integration expressed in different readings of each of the components of this description (i.e., what does ‘distributed’ imply? How is it ‘European’? What is a ‘research infrastructure’, and what is it for?). I conclude by pointing to the ways Europe, science, and European integration are framed in the mundane organisational and discursive components that make a research infrastructure.

## European research policy and the establishment of BBMRI-ERIC

Research in STS and the history of science and technology has indicated how sociotechnical collaboration across borders has played a central role in the making of multiple, overlapping, and yet incongruous spaces called ‘Europe’ since the nineteenth century. These forms of Europe-making included, for example, the building of cross-national (transportation and energy) infrastructures, the establishment of international expert organisations, the development of shared standards, and the institutionalization of scientific collaboration (Badenoch & Fickers, 2010; Barry, 2001; Krige, 2006; Trischler & Kohlrausch, 2014). These initiatives largely preceded or happened in parallel to the formal economic and political integration processes that started around the middle of the twentieth century. Like these forms of integration that would eventually produce the European Union (EU) as we know it today, scientific and technological integration was not linear. Both happened in waves that included episodes of disintegration as well as closer collaboration (Patel, 2018; Von Hirschhausen & Patel, 2010). Within the context of the European Communities and later the EU, cross-national scientific collaboration was only taken up as an area of active intervention from the 1970s onwards. Reform proposals in the 1980s formally brought research policy within the remit of European institutions. Since the 1990s, the EU has gradually expanded its activities aimed at advancing European scientific integration. It moved from research funding (under the EU framework programs) to the idea of an integrated European Research Area (ERA) with a central role for research infrastructures. This notion itself gained prominence in the first two decades of this century (Cramer et al., 2020), bookmarked by the establishment of an intergovernmental European Strategy Forum for Research Infrastructures (ESFRI) in 2002, the publication of its first Roadmap prioritizing certain infrastructures in 2006, and the introduction of the dedicated legal framework ERIC in 2009.

BBMRI–ERIC developed almost exclusively under the auspices of these formalized procedures for establishing transnational European research infrastructures (Vuorio, 2017). Calls for establishing a network of European sample and data collections for medical research largely developed within working groups preparing the 2006 ESFRI Roadmap. The working groups that proposed a European biobanking network did so based on a vision of the benefits of further European harmonization and integration and the risks of continued technical and regulatory fragmentation. They pointed to the scientific potential for large scale biomedical research if the many existing collections of samples and data—whose quality was painted as a particular European strength—could be made accessible and interconnected (European Science Foundation, 2008; Yuille et al., 2008). Adopting this vision of a biobanking network’s potential contribution to larger biomedical research endeavours, the first ESFRI Roadmap included a proposal for a ‘European Bio-banking and Biomolecular Resources’ infrastructure. The proposal thereby became eligible for dedicated EU funding for developing a blueprint for the infrastructure, which was subsequently used for financing the ‘Preparatory Phase’ of what was then known as BBMRI. The project ran from 2007 until 2011, resulting in a ‘Business Plan’ proposing an organizational outline for the infrastructure (Zatloukal et al., 2012). In the meantime, the European Commission had introduced the ERIC framework, which was subsequently adopted as the preferrable structure for governing BBMRI going forward. In 2013, BBMRI was only the second European research infrastructure receiving ERIC status. Below, we will return to certain episodes in the history of what has since been called BBMRI–ERIC to illustrate different meanings people attributed to them vis-à-vis their own ideas of what a distributed European research infrastructure is.

**Figure 1.**
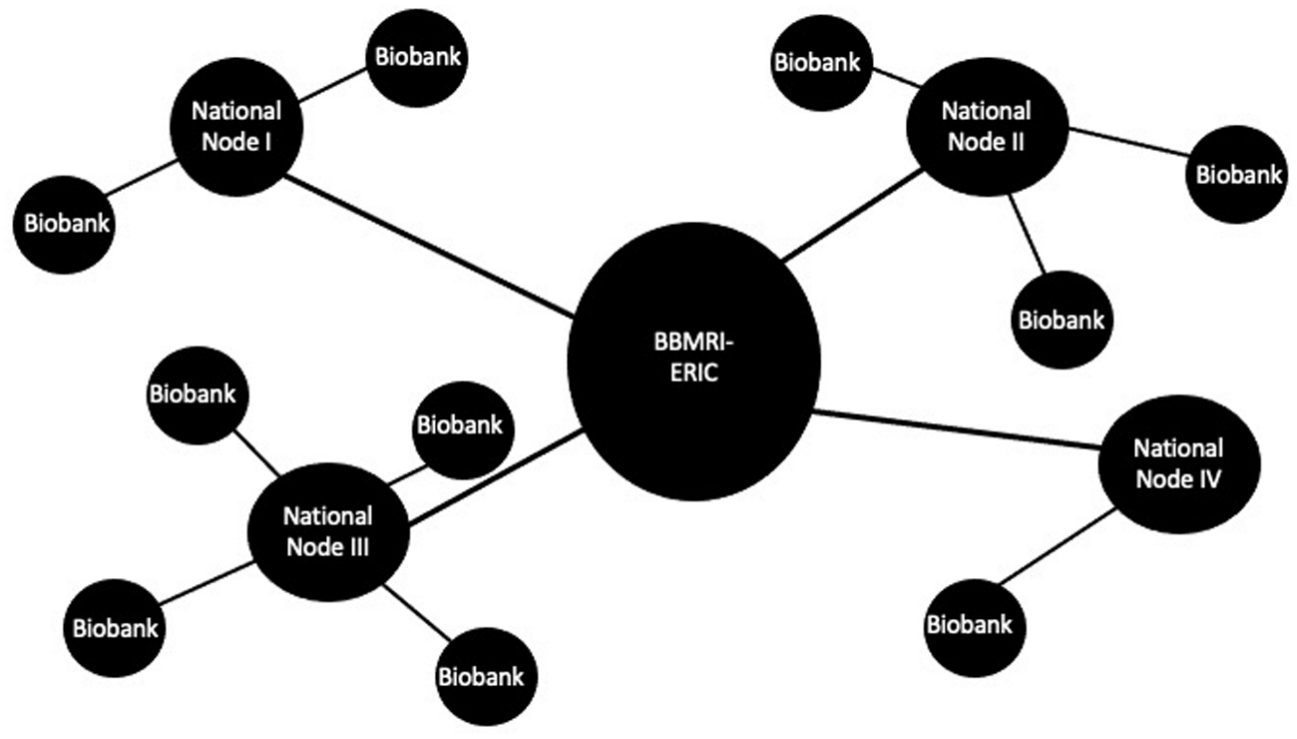
The distributed ‘hub-and-spoke’ organizational structure of BBMRI–ERIC. Figure by the author.

The description of BBMRI–ERIC as a distributed European research infrastructure reflects important aspects of its organizational configuration (see Figure 1). This configuration was largely developed during the Preparatory Phase, although some elements are specific to the ERIC framework. A central aspect of BBMRI–ERIC is that it is essentially conceived as a network of networks. In accordance with the ERIC regulation, nation-states rather than individual biobanks are the ‘members’ of the infrastructure. Membership must include at least three countries, and half of all members must be EU member states, including the infrastructure’s ‘host country’ (which for BBMRI–ERIC is Austria). Representatives of the Member States (usually from science or health ministries) make key decisions, such as approving the annual budgets or work programs within the ‘Assembly of Members’. At the same time, Member States are obliged to establish a national biobanking network, which is represented within BBMRI–ERIC by a so-called National Node (which includes a scientific coordinator who is usually not the same person as in the Assembly of Members). In addition to the National Nodes, BBMRI–ERIC has a central executive office, commonly known as ‘headquarters’, which is located in Graz, Austria. The headquarters is led by the Director-General and employs a number of people who coordinate the activities of the National Nodes, develop and maintain so-called Common Services in IT and ELSI, and perform central activities in areas such as quality management and stakeholder engagement.

While we will see how specifics of the infrastructure’s development and organization contribute to how European scientific integration is imagined in BBMRI–ERIC, broader visions regarding its benefits to science and to Europe abound, too. In journal publications introducing the infrastructure, staff members emphasize both its character as a *European* infrastructure and the benefits the infrastructure can generate *for Europe*. Articles describe BBMRI–ERIC as ‘by nature a distributed infrastructure […] a pan-European hub-and-spoke distributed structure’ (Litton, 2018, p. 234) that forms ‘a gateway to the existing comprehensive collections of population-based and disease-oriented samples from different (sub)populations in Europe’ (Mayrhofer et al., 2016, p. 381). BBMRI–ERIC’s distributed character is also presented as a source of potential for biomedical research in Europe. It allows BBMRI–ERIC to be positioned as ‘…not just any research infrastructure, [but] a critical infrastructure for health in the EU’ that gives Europe ‘a striking advantage’ due to biobanks’ ‘potential for a substantial impact on the economic growth and improvement of healthcare’ (Litton, 2018, p. 240). Such claims reflect how the need for EU Member States to maintain biobanks is discursively produced. Connecting biobanks across Europe supposedly ensures that ‘we are able to harvest [their] benefits for European citizens in the future’ (Litton, 2018, p. 240). This argument links BBMRI–ERIC’s distribution throughout Europe, and its ability to capitalize on existing collections, to the salient science policy rhetoric that biomedical research will generate ‘health and wealth’ (Gardner & Webster, 2016) for all Europeans.

Previous social science engagements with BBMRI–ERIC have pointed out how such visions intersect with the particular sociotechnical configuration of the infrastructure. Lauss (2009, p. 250), for example, placed BBMRI (as it was then known) in a broader context and observed how ‘the (self-)governance capacities of the scientific biobank community become intertwined in political macro strategies of European integration and the “knowledge based economy”.’ He argues that BBMRI–ERIC can be understood as ‘a materialization of the process of European integration’ (Lauss, 2009, p. 265). Such materialization can, for example, be found in efforts to develop a common technical language for describing biobanks and their collections. Tamminen (2015, p. 2) similarly argues that such efforts seek ‘to provide an informational backbone for the large-scale biomedical infrastructure platform envisioned by European research policy.’ Scientific and policy actors seek to bring together ideas of European and scientific collaboration, with potentially significant implications for the organisation and practices of science in Europe. Along these lines, the criteria and procedures that BBMRI–ERIC has developed to work towards its core aims of improving access to and sharing of materials shift biobanks’ focus from sample storage to sharing. Due to the central role of the infrastructure in the European biobanking landscape, such shifts furthermore resonate beyond formal BBMRI–ERIC membership (Argudo-Portal & Domènech, 2020). Such observations point to the distinctive role of this particular research infrastructure in shaping ideas of what ‘science’ and ‘Europe’ are.

## Conceptualizing European research infrastructures as relational, experimental and promissory assemblages

Although the perception of biobanks and biobanking networks as ‘infrastructure’ gained prominence through European research infrastructure policy, the label has grown ubiquitous beyond that specific context. Biobanks are described as infrastructures with reference to their importance for enabling medical research (Hewitt & Hainaut, 2011; Knoppers & Hudson, 2011; Meijer et al., 2012). They can broadly be defined as ‘structured collections of biological samples and associated data stored for the purposes of present and future research’ (Parodi, 2015, p. 15). As such, they are considered valuable to various research fields (Hewitt, 2011; Zielhuis, 2012), for the ‘translation’ of laboratory findings into clinical applications (Murtagh et al., 2011; Riegman et al., 2008), as well as for economic growth, social cohesion and (national) identity (Busby & Martin, 2006; Tutton, 2010). Biobanks are associated with growing scales of biomedical research, since their potential to be of value is largely attributed to their ability to provide large numbers of research participants. Such large numbers are in turn considered necessary for understanding the intricate dynamics of health and disease (risks) in (post)genomic research (Asslaber & Zatloukal, 2007; Simell et al., 2019). This view of biobanks as infrastructures for large scale research logically extends to joining together multiple biobanks in even larger networks. Such networks can ‘increase the power of research by allowing access to a larger pool of suitable samples, or to share expertise in scientific and technical aspects of biobanking’ (Shickle & Griffin, 2009, p. 1).

Despite the widespread descriptions of biobanks as infrastructure, and its more generally increasing prominence in research policy contexts, the designation ‘research infrastructure’ is rarely defined. Cramer et al. (2020) argue that research infrastructures are ‘consecrated’ in the European policy context, defined through the act of designation by EU institutions rather than on the basis of explicit criteria. Policy analysts have consequently argued that research infrastructures ‘do not constitute an organizational field or category of analytical relevance’ (Hallonsten, 2020, p. 618). This line of thinking suggests that *research infrastructures* is not a conceptually useful notion, despite the various ways in which the notion circulates and affects how research is organized and visions of its societal value are articulated.

By contrast, research in science and technology studies (STS) and anthropology has conceptualized infrastructures as pivotal to a wide variety of forms of circulation and exchange in a range of social domains, including research. Slota and Bowker (2017), for example, have described infrastructures in terms of the technical, organizational and social work required to build and operate them, which in turn facilitates other forms of work. Yet, infrastructures do not only support other forms of work, they are also imbued with meaning and promises of improved futures (Anand et al., 2018). Social science work on infrastructures has therefore approached infrastructures as assemblages of social and material standards, classifications, procedures and other elements that structure various forms of circulation—of people, things, and meanings. The growing STS literature on infrastructures in their many sociotechnical forms includes three conceptual strands that I consider of particular value in tracing how scientific and European integration are materialized and narrated in European research infrastructures. These include descriptions of infrastructures as relational, experimental and promissory assemblages.

First, in their study of the integration of information systems in biology, Star and Ruhleder (1994, p. 253) propose to think of infrastructures as ‘fundamentally and always a *relation*, never a thing’. Their perspective implies that social and material relations change with the establishment, maintenance, transformation, and degradation of infrastructures. Along these lines, Vertesi (2014) describes how connections between multiple, largely incompatible scientific infrastructures require the (temporary) stitching together of the material ‘seams’ between different systems. Research on the growing importance of exchange and circulation of materials and data in the life sciences has also shown how standardization and coordination of data formats is not only a matter of building technical infrastructure, but also of shared social relations across research sites (Leonelli, 2016; Tupasela, 2010). These examples show how the making and interconnecting of research infrastructures includes the creation of new socio-material relations.

Second, new relations emerging through research infrastructures can have substantial, often unanticipated, effects on scientific fields. Referring to this broader transformative potential of infrastructures, Jensen and Morita (2015, p. 82) propose to think of infrastructures as ‘at once material and *experimental*’ (emphasis added). Infrastructures create ‘novel configurations of the world and its elements’ (Jensen & Morita, 2015, p. 84), which can have far-reaching consequences for the epistemic, technical, organizational and experiential contours of scientific disciplines and practices. In their study of new computational infrastructures in ecology, for example, Jackson and Barbrow (2013) illustrate the effects of technical transformations on the discipline. Parts of ecology shifted from a field science to a desk science, changing relations to particular objects and places. Both the temporal and spatial configuration of ecology changed, creating a new kind of scientific discipline through the introduction of new infrastructural configurations.

Third, the design, planning and building of infrastructures is also enmeshed with *promises* of social progress. Larkin (2013, p. 333) has emphasized this dimension by observing how infrastructures ‘also operate on the level of fantasy and desire’, incorporating visions of desirable social futures. Both particularities of infrastructural design and construction, as well as the ways diverse social actors engage with an infrastructure reflect such promises (Appel et al., 2018). As infrastructures for storing materials that may realize their value in the future, anticipated futures often form important motivations for biobanking projects. In his work on the Icelandic Health Sector Database, for example, Fortun (2008) emphasized how promises are central to the building of infrastructures for genomics research. Infrastructures are thus built as vehicles for realizing promises of social progress, including scientific, social, and economic benefits.

Conceptualizing research infrastructures in terms of relations, experimental sociotechnical realities, and promises allows for a fine-grained analysis of how scientific collaboration and European integration get jointly imagined and materialized. Such analysis is attentive to the different meanings infrastructural formations may entail for different actors. This attention to the interpretive flexibility (Bijker, 1995) of infrastructural formations suggests that research infrastructures generate heterogeneous formations of European integration and scientific collaboration. In what follows, I trace both the organisational contours of BBMRI–ERIC and the discursive representations and interpretations of how this infrastructure is said to serve European scientific integration. In doing so, I will show how perennial contestation over the meaning of ‘Europe’ itself (Pagden, 2002) is interwoven with disputes over the question what the purpose of research infrastructure is.

## Analysing organisational and narrative configurations of European research infrastructures

To analyse the meanings attributed to BBMRI–ERIC and how these meanings provide insight into the heterogeneous and contested character of European scientific integration, I draw on Bowker and Star’s (1999) methodological proposition of infrastructural inversion. They present this proposition as a way to interrogate the taken-for-granted character of infrastructures and to identify the particularities of infrastructure’s material and political constitution. Discussing various examples of classification infrastructures, Bowker and Star show how particular technical conditions, enmeshed with political and moral priorities, shape the configurations of these infrastructures, often with lasting effects. I build on this methodology by using different perspectives on BBMRI–ERIC as ‘inversions’ of not only the infrastructure’s organizational configuration, but primarily of the narratives that articulate its significance for European scientific integration. To explore these narratives, I also draw on Czarniawska’s (1998, 2004) work on narrative analysis. She focuses on how narratives represent social realities and their development through plots that aim to provide explanations for, and logics to, a particular sequence of events. I hence depart from studying infrastructural inversion with a focus on materiality and operational practice (which would pursue substantially different questions when applied to a networked, largely ‘virtual’ infrastructure such as BBMRI–ERIC), to consider how this particular infrastructure’s organizational and rhetorical positioning illustrate key aspects of and disagreements over European scientific integration.

For my reconstruction of how European and scientific integration are narrated and disputed in BBMRI–ERIC, I use a range of materials that represent the infrastructure in different ways. I provide an overview of these materials in Table 1. They include documents, observations of public performances by staff members and interviews. While BBMRI–ERIC is presented in similar ways in many of these materials, they each serve different purposes in representing BBMRI–ERIC.

**Table 1.** Overview of research materials analysed in this paper.

Materials		Reference in text
Documents	*Publications*	Author-date reference
	PubMed search “BBMRI”; 101 results, 38 selected based on title/abstract	
	*Work Programmes*	Author-date reference
	2015-2020	
	*Annual Reports*	Author-date reference
	2014-2019	
	*Biobank Europe Magazine*	*Biobank Europe* + issue number (e.g., *Biobank Europe* 4)
	7 issues, published 2015-2017	
	Others	Author-date reference
	e.g., Business Plan, Statutes, Vision Paper	
Observations	*Europe Biobank Week Conferences*	EBW+year (e.g., EBW, 2016)
	2016 (Vienna), 2019 (Lübeck), 2020 (online)	
	Webinars	Webinar + date (e.g., Webinar, October 2019)
	Six webinars between October 2019 and May 2020; focused on IT (2), ELSI (1) and Covid-related services (3).	
Interviews	*13 semi-structured interviews with BBMRI–ERICstaff members and key actors.*	Some respondents chose to be identified by name; others are characterized according to their involvement with BBMRI(-ERIC), as BBMRI or HQ (meaning BBMRI–ERIC‘headquarters’) staff members and National Node coordinators (e.g., HQ staff member 4).
	Conducted between October 2019 and December 2020.	

In various *documents* published by the infrastructure itself, it seeks to present a formalized version of what it is and does, often in quite some technical, regulatory, and organizational detail. These documents include regular publications such as annual reports and work programs, as well as a series of *Biobank Europe Magazines* the infrastructure briefly (2015-2017) published. They further include more singular documents that map out the infrastructures structure and tasks such as the business plan (2012), statutes (2013) and a vision paper (2018).

*Public performances* by staff members seek to present the infrastructure and explain its activities to audiences such as biobanking professionals, the broader medical research community, and policymakers. They include journal publications that provide information about the infrastructure, webinars, and presentations at international conferences, especially in the fields of IT, ELSI and quality management, as well as in response to the COVID-pandemic.

Finally, I conducted *interviews* with current staff members, people involved in earlier stages of the infrastructure’s development, and others working with BBMRI–ERIC in various capacities. With these semi-structured interviews, I gathered perspectives from thirteen individuals on the origins, development and current operations of BBMRI–ERIC. Interviews were conducted between October 2019 and December 2020, some in-person and others by phone or online. All respondents were given a project description and signed a consent form. Two respondents opted out of verbatim citation from the interviews; four others requested to be identified in publications; the remaining seven individuals have been anonymized. Since the community of people working closely with BBMRI–ERIC (and especially the staff at the central administrative office) is relatively small, I present minimal descriptions of individual respondents here to avoid identification.

To process and analyse these materials, I used an open coding approach that was primarily descriptive, but followed an ‘in vivo’ approach with regard to terminology widely used by the actors themselves. In a second step, I sought to identify patterns in descriptions of BBMRI–ERIC and the coherence between elements of these descriptions (Saldaña, 2009). I used the tenets of abductive analysis (Tavory & Timmermans, 2014) to trace the heterogeneous meanings attributed to BBMRI–ERIC as a distributed European research infrastructure as well as to the constitutive elements of this designation. In particular, the abductive approach served to bring my literature-based conceptualization of research infrastructures into conversation with the oft-used, but often only implicitly defined references to this notion in the materials. This approach can help understand how research infrastructures have become salient crystallization points for imaginaries of science as an engine of European integration.

## BBMRI–ERIC as a heterogeneous distributed European research infrastructure

BBMRI–ERIC reports its ‘ultimate goal [is] to facilitate access to biological resources as well as biomedical facilities and to support high-quality biomolecular and medical research’ (BBMRI–ERIC, 2015, p. 3). This central aim brings together a set of advantages and ambitions associated with interconnecting European biobanks. It builds on the idea that wider access to and sharing of resources such as samples and data will enhance biomedical research. It advances the idea that European welfare states have a long tradition of high-quality storage of human biological materials and associated data. It also reflects the belief that better use of existing collections holds potential for research that would take decades to build up when establishing biobanks de novo. The ‘ultimate goal’ of BBMRI–ERIC thereby encapsulates why this network has been imagined and configured as a ‘distributed European research infrastructure’. However, the form and purposes of these characteristics have been interpreted in various ways as BBMRI–ERIC was being established and developed. In this section, I therefore explore subsequently how its distributed character, its European identity, and its role as a research infrastructure take on heterogeneous forms in both actors’ narratives and the socio-material configuration of BBMRI–ERIC itself.

### Distributed samples and intergovernmental governance

The designation of BBMRI–ERIC as a distributed European research infrastructure refers both to its governance structure, which allocates a central role to the Member States, and the continued decentralized storage of samples and data. A former administrator illustrates the former by emphasizing how, from the beginning, ‘not the individual biobanks [are] part of BBMRI, but […] it’s always a national network of biobanks. We wanted to have a strong national coordination in each of the countries’ (BBMRI staff member 2). On the other hand, Jan-Eric Litton, who was the first Director-General of the infrastructure after it received ERIC status, emphasized in an interview how ‘BBMRI–ERIC by itself doesn’t own any samples. It is distributed by its soul’. To him, the distributed soul of the infrastructure is related to its scientific aim, which is—in the words of one speaker at the online Europe Biobank Week conference in 2020—to avoid that biobanks become ‘biohoards’ that only store and do not distribute or share their materials. The importance of sharing is, in turn, related to promises of large-scale research, with ever increasing numbers of participants, which is supposed to be more productive (Davies et al., 2013) and requires the combination of different collections.

This view of the promise of biobanking is particularly future-oriented and expressed in terms of research that may become possible later if samples and data are collected now. A medical professor in a BBMRI–ERIC member state explained the promise of biobanking along these lines:

One thing you can do is putting all instances of a disease together and look at, who survived, who didn’t. That’s instructive and very good research, but it remains a kind of burning-house research. When you go and look in a biobank of a hundred thousand people and you have taken serum samples over twelve, fifteen years, every five years, or two years […] And then a certain number of people […] you look at them for twelve years, and around 180 people have pancreatic carcinoma. Later. Then you go back to the samples of [those] people. (National Node coordinator 1)

Those samples can help in finding commonalities between people with pancreatic carcinoma, which may explain why a particular sub-group developed the disease. Such research requires large numbers of participants to find statistically significant differences between them.

This belief in the scientific potential of having access to large numbers of samples and their associated data extends beyond individual biobanks. As one administrator who was involved in the set-up of BBMRI early on illustrates, new technological capabilities informed new research questions, which in turn required more research materials. He presents biobanking networks as a continuation of the Human Genome Project:

About that time, it started that you could start to analyse hundreds and thousands, if not ten-thousands of samples in one round, and then people, or scientists realized that each biobank would have different formats [etc.], so there was a need to have a coordinated approach in standardization, in harmonization, in further development, and also in providing access. It’s easy if you need a few hundred of samples […]. But when you today want to analyse a hundred thousand or two hundred thousand or a million, not a single biobank has those samples, so, you will need to negotiate with several, if not dozens of different biobanks. (BBMRI staff member 2)

For biobanking professionals in Europe, the potential of doing larger-scale research motivated new relations between different European biobanks.

The scientific promise of networking biobanks emerged in close temporal proximity to the ambitions of EU research policymakers to establish European ‘research infrastructures’. From the beginning, these were not only imagined in terms of collaborative scientific relations, but also as explicit contributions to a European integration agenda. The first ESFRI Roadmap, which was published in 2006, makes the case by arguing that ‘Research Infrastructures of pan-European relevance provide unique opportunities […] for European capacity building’ (European Strategy Forum on Research Infrastructures, 2006, p. 10). It continues by emphasizing how research infrastructures have historically contributed to the strength and international visibility of European research, while warning that the EU might lose its international leadership position in the absence of further investments. ESFRI therefore makes the case for establishing new research infrastructures, which also ‘afford the opportunity to aid European integration’ (European Strategy Forum on Research Infrastructures, 2006, p. 15). References to a classic scenario of things Europe is good at, which may be at risk if no further investments are made, thus support the scientific and political promise of research infrastructures.

Recalling his involvement in the development of a proposal for the Roadmap that would eventually become BBMRI–ERIC, a researcher from a Western European country pointed to the nuclear research organization CERN as a model for the ESFRI proposals. As he explained, CERN provides an example for the logic behind research infrastructures, which is for ‘[t]hings that are too expensive for one country, which you then want to do together’. He continued by sketching how the idea for a biobanking infrastructure became possible because, ‘now, for the first time, next to the ships going through polar ice to take biological samples, or other things, they added a biomedical and social chapter. And the biomedical and social chapter, there was a clinical commission and a genomics commission’ (National Node coordinator 1). Both of these commissions, in his recollection, ended up with fairly similar proposals to establish a European biobanking infrastructure, joining existing sample and data collections together.

The use of examples such as CERN and polar research vessels illustrates how for biomedical researchers, thinking of collaboration in terms of infrastructure was highly experimental. Another leading contributor to the development of BBMRI recalled how the idea to develop a research infrastructure for the life sciences formed ‘completely new territory’. He, too, drew on an understanding of research infrastructures as a phenomenon common to other fields working with large machinery. He explained that ‘it was relatively unclear, what does a research infrastructure in the life sciences mean? Because in astrophysics, there was a tradition [where it] was clear, okay, a telescope is a telescope, right?’ (BBMRI staff member 3). Although this point of view probably underestimates the complexity of building a European telescope, it draws a distinction between single-sited infrastructures built around large experimental machinery and distributed approaches that are considered more suitable for the biomedical sciences. Despite this ambiguity, the idea of developing a European research infrastructure involving biobanks was included in the first ESFRI Roadmap. On that basis, it was eligible for European Framework Program funding, which was used to finance the Preparatory Phase project with the aim of developing an outline for a European biobanking infrastructure.

The main outcome of the Preparatory Phase was the Business Plan, a blueprint for how BBMRI would continue to run. Echoing the ESFRI Roadmap, it described a distributed infrastructure that ‘builds on existing collections, complemented with innovative components’ in which ‘[a]ll resources will be integrated into a pan-European distributed hub-and-spoke like network, and will be properly embedded into European scientific, ethical, legal and societal frameworks’ (Zatloukal et al., 2012, p. 15). It emphasized how the scope of work would extend beyond the scope of individual Member States. This was to be achieved by enhancing the interoperability of European biobanks to address prominent medical research questions. BBMRI–ERIC was framed as ‘an effective measure to reduce fragmentation of the European landscape and increases competitiveness through collaboration’ (Zatloukal et al., 2012, p. 15), which was imagined to have both a scientific and a socioeconomic impact. The Business Plan thereby outlined the relations between the infrastructure, member states, and individual biobanks that would be part of the experimental configuration of a ‘pan-European distributed hub-and-spoke’ infrastructure. Such descriptions helped articulate the distinct (European) promises this initiative would hold.

Alongside the development of an outline for BBMRI, the European Union continued to experiment with novel forms of European scientific integration. Largely in parallel to the BBMRI Preparatory Phase and similar projects to advance research infrastructures sketched out in the ESFRI Roadmap, the European Commission (the EU’s executive branch) developed a regulatory framework called European Research Infrastructure Consortium (ERIC), which was adopted in 2009. Providing a blueprint for how to organize and govern cross-national European research infrastructures, it was soon considered to be the most desirable institutional format for BBMRI beyond the Preparatory Phase. As Eero Vuorio, who was executive director of the Preparatory Phase, recalled in an interview, ‘Scientists are daydreamers, and they can come up with all kinds of nice structures. And then they go to their home ministries, and there is no daydreaming in the whole ministry at all’. His opinion was corroborated by a member of BBMRI’s administrative staff, who used somewhat less picturesque terms to argue that ‘when it comes to business planning, service delivery, scientists typically fail’ (BBMRI staff member 2). The ERIC framework provided a ready-made governing format, one that relied on the commitment of an infrastructure’s member states and thus created new relations within and between European countries around the institutionalization of scientific integration.

Yet the ERIC framework was itself an experiment. It only gained real substance with the application of several research infrastructures. The ERIC framework was supposed to provide a single legal structure for infrastructures across all scientific disciplines and research traditions. However, the framework’s ‘plasticity’ (Moskovko, 2020) that allowed it to operate across scientific disciplines restricted its value for specific fields. Jan-Eric Litton, for example, considered the value of the framework for biobanking to be limited, since ‘the ERIC status doesn’t care about […] sharing samples across borders’ (see also Reichel, 2015; Reichel et al., 2014). The ERIC framework thus contributes to the recognition of certain collaborative techno-institutional formations as designated European research infrastructures but may, as such, not provide much support for the distributed mode of work of European biobanks.

BBMRI–ERIC is thus a distributed European research infrastructure in various ways—both in terms of its aims to join biobanks distributed around Europe and in its governing framework that puts an emphasis on what one medical researcher described as the role of member states as ‘owners’ of the network (BBMRI staff member 3). Nevertheless, some people see the proactive role adopted by the central ‘headquarters’ in Graz as in contradiction to the infrastructure’s distributed character. The former coordinator of the French National Node, Georges Dagher, for example, indicated his belief that BBMRI–ERIC is too preoccupied with centralizing activities. He emphasized that he considers the infrastructure’s operations not distributed *enough*. As he put it, ‘BBMRI–ERIC is not only the coordination in Graz. BBMRI–ERIC is the whole infrastructure […]. It is the 500 or 600 biobanks in the countries.’ His perspective points to a paradox in BBMRI–ERIC between the importance attached to distribution and the work done to make it work and act as one single infrastructure.

### European strengths and cross-national differences

BBMRI–ERIC is often described as a distributed *pan-European* research infrastructure, yet the question what makes it (pan-)European is not straightforward. Not all countries that are part of the European Union—let alone those that may reasonably be considered ‘European’—have joined. However, rather than suggesting some kind of European whole, interview respondents suggested that the geographical spread of members and diversity in their size and biobanking expertise made the infrastructure legitimately ‘European’. A senior medical researcher from a Northern European country, who was part of one of the ESFRI working groups, saw strength in diversity. He presented this viewpoint in terms of a largely speculative list of national differences in expertise:

Sweden has traditionally been very good in, particularly, the oncological biobanks and IT. And we [in country X] do this very well, and in France they do something else very well, and in Finland they do something else very well, and in Estonia they do still something else very well, and, yeah, you are actually trying to particularly tie those strengths together. (National Node coordinator 1)

While not very specific on the strengths of individual countries used as examples (which, not coincidentally, have an established reputation in the context of biobanking), he suggested that mutual benefits could be drawn from cross-border collaboration. Maintaining the central importance of the member states, BBMRI–ERIC itself declares that it ‘bears on the strengths of all its members large and small’ (*Biobank Europe* 3, p. 6). It describes the National Nodes as ‘powerhouses’ that ‘coordinate the biobanks within their countries while actively contributing to research and growing our sample collections’ (BBMRI–ERIC, 2019). Membership of BBMRI–ERIC is thereby presented as beneficial for biobanking at both a national and a European level.

Considering the promise of a joint European biobanking infrastructure from the point of view of the member states, Lukasz Kozera, a Polish national employed at BBMRI–ERIC headquarters, recalled some of the discussions in his home country before joining:

Some of them [Polish biobankers] heard about [BBMRI–ERIC] some of them did not. And they were doing a lot of research of, like, how is it actually working, what kind of implications will it have for our activity. Whether they will be obliged, let’s say, to introduce their quality schemes, or, for example, you know, any kind of transformation of data, any kind of common IT tools. So, there was a huge fear at the beginning, how it’s going to look like if we join BBMRI–ERIC. But step by step we were introducing them to the whole idea and showing them pros and cons of being a part of that huge network.

Differences between (potential) member states may also prompt individual countries to join BBMRI–ERIC or not. A different official used the examples of Spain (which was not a member at the time, but has since joined) and Bulgaria to illustrate:

[The wish to join,] that’s mostly driven by science, so if there is a community of scientists, usually they approach us and then we approach the ministry, that happened for Spain for example. Again, stupid situation, they had one of the most advanced an organized networks of biobanks in Spain, it’s super good, but Spain doesn’t want to pay for, you know, […]. In other countries, it happens the other way around, Bulgaria—they had a change in the government, new minister, they had the [EU] presidency, they wanted a high-level commitment on research, and of course research infrastructures is a great way to go ahead. (HQ staff member 4)

National networks and individual biobanks are also an important point of reference for the promises that building an infrastructure for biobanks in Europe entails. While many publications and strategy documents argue for the benefit for science, health, and the economy in Europe as a whole, BBMRI–ERIC also claims to be beneficial for individual Member States. It has, for example, tried to quantify some of the advantages of membership in financial terms, for example by listing how much money each Member State receives out of EU-funded projects in which BBMRI–ERIC participates (BBMRI–ERIC, 2017a, 2018b). Especially in countries that joined BBMRI–ERIC more recently, many of which were formerly located in the Soviet bloc and have also (relatively) recently joined the EU, the possibility of adapting to and learning from other European countries is presented as a key motivation for joining. One example is the virtual poster that presented biobanking networking and standardization efforts in Lithuania at the 2020 online *Europe Biobank Week* conference, shortly after the country joined as an observer.

However, membership in BBMRI–ERIC is not only a matter of national research networks—known within the infrastructure as ‘National Nodes’. As a formal European legal entity, the ERIC also involves government institutions—usually research or health ministries—who provide funding and govern the infrastructure. As a former headquarters official explained:

There are really two strands within BBMRI […]. There are the ministries who say: ‘we pay, we pay you for services. We want you to help biobanks in Europe’. The National Node directors say: ‘you have to help me get projects, so I can do research’. Those two priorities, even within a country, are completely different. (HQ staff member 2)

This interview respondent thus described one of the challenges for BBMRI–ERIC as striking a balance between the different priorities both at the national and European level.

Both the relations between various member states and the different scientific and political priorities within them are negotiated in the infrastructure’s annual budget. The distribution of contributions to the infrastructure and purposes for which financial resource are used are one way by which European scientific integration gets made. The so-called core budget is calculated based on the Director General’s proposed Work Programme for the following year and negotiated with the Assembly of Members, in which all Member States are represented. Member States contribute proportional to the size of their populations and economies, with each contribution consisting of a set amount (€ 25.000 for countries with more than 3 million inhabitants, €20.000 for those with less) and a flexible portion. This part is based on the remaining amount the Assembly of Members has agreed to, divided by each country’s gross domestic product (GDP) as a percentage of the aggregate GDP of all Member States. For the year 2020, for example, this means that Malta committed to paying €21.297,02, whereas Germany, a much larger country with a higher GDP, was committed to €396.221,36 (BBMRI–ERIC, 2020). Overall membership contributions for 2020 were €1.705.833,53, with host country Austria adding around €100.000 more and about half a million consisting of other earnings.

BBMRI–ERIC’s core budget (which does not include income from EU project funding) can be read as an instrument that makes the infrastructure European in particular ways. On the one hand, interview respondents indicated that the consideration of population size and economy contributes to the diversity of membership, allowing smaller and poorer countries to join along the large and rich ones. On the other hand, the budget provides insight into how the relations between centralized European and national commitments are imagined, at least in financial terms. Several interview respondents lamented how the ‘core’ budget is not sufficient to cover the infrastructure’s basic operation costs. The budget therefore needs to be structurally complemented with project funding (see also BBMRI–ERIC, 2016, 2017b). Moreover, BBMRI–ERIC officials express frustration with the Assembly of Members’ unwillingness to adapt the budget to changing circumstances. One official, for example, described how he learned that the budget follows members’ willingness to pay when France terminated its membership:

So, we consciously adopted a resolution in the Assembly, to proportionally reduce the budget. That is, it is really troublesome that it is non-negotiable to increase the budget. (HQ staff member 2)

This person further added that the infrastructure therefore does not have the means to develop its activities further.

By contrast, another former staff member argued that the particular structure of BBMRI–ERIC implies that the budget should be considered in different terms:

[This budget] is the European coordination. If you would include the expenses of all the [at the time] twenty-one networks and the costs of all the several hundreds of biobanks participating, very likely you come much closer to what you know from single-sited, big physics research infrastructures, which is in the magnitude of somewhere between half a billion and a billion a year. (BBMRI staff member 2)

His suggestion that the overall budget of BBMRI–ERIC should be seen as including national and institutional funding as well indicates how the infrastructure is European both in terms of its central coordination and through the various ties between largely independent member states.

The contributions of national networks to the European infrastructure go beyond the distribution of financial commitments at the national and European level. National Nodes also contribute to the experimental making of European biobanking assemblages by contributing in various ways to European coordination and harmonization efforts. Thematic sessions at various events such as the *Europe Biobank Week* conference, for example, include presentations of national projects developing quality management or data exchange procedures that are often extended to BBMRI–ERIC as a whole. Large parts of the Directory and Negotiator IT-platforms, which allow searching for samples in member biobanks and negotiating access to these samples, respectively, were developed in German projects (interview, National Node coordinator 3).

While many of these examples support the notion that networking diverse countries contributes to the promise of European collaboration, there is a widespread sense that biobanking in Europe is characterized by asymmetrical relations. Preparatory Phase director Eero Vuorio, for example, referred to ‘sort of a North–South gradient in this area,’ where ‘[a] tradition of collecting samples particularly is a North-of-the-Alps-phenomenon.’ The supposed ‘European’ tradition of sample collecting thus gets narrowed down to (predominantly) Scandinavian countries, where a discourse of the future potential of the welfare state’s past efforts in collecting population data and materials finds wider resonance (Cool, 2016; Tupasela et al., 2020).

BBMRI–ERIC activities not only seek to overcome such asymmetries, but occasionally also reproduce them. The project BBMRI–LPC (for large population cohorts), which started towards the end of the preparatory phase and continued until the early days of BBMRI as an ERIC, aimed to ‘[facilitate] transnational access to samples and health data in the large European follow up studies, thereby increasing their utilization for health research’ (BBMRI–ERIC, 2015, p. 40). It included the creation of a catalogue of existing population cohorts, which included ‘strong representation of Scandinavian countries, the UK and the Netherlands, which, thanks to their registers and population cohorts, have very good resources for epidemiological research’ (Kuhn et al., 2016, p. 388).^1^ For Eastern European countries, the project sought to identify the mere existence of biobanks, which indicates how behind the pan-European curtain, the *European* tradition of collecting samples does in fact only seem to be pronounced in a small subset of countries.

Moreover, the predominant role of member states in BBMRI–ERIC raises concerns regarding in how far it can be considered a distributed *European* infrastructure. One staff member considered the European character to be secondary to member state interests:

these infrastructures are not really, I mean, they are European in the sense that […] the regulation facilitates the creation of this multi-national infrastructure, but as a matter of fact, they’re not, if you’re not, if your country doesn’t pay the membership fee, you’re not going to access my infrastructure […]. It’s still driven by national interests. It looks very European, but it’s connected and it boils down to which country is interested in what. (HQ staff member 4)

This person saw the central role of member states as being in opposition to what a truly European infrastructure would look like.

### Research Infrastructures as science or service

In spite of the many differences between member states and individual biobanks, all members need to accept certain criteria to participate in the distributed European *research infrastructure* BBMRI–ERIC. Participating biobanks need to accept the Partner Charter. This document includes the obligation ‘to foster scientific excellence, guarantee interoperability, and compliance with ethical and legal requirements’ (BBMRI–ERIC, 2018, p. 6) through the implementation of best practice guidelines, the development of an access policy, etc. Member States, in turn, are instructed in Article 4 of the infrastructure’s statutes to establish a National Node that maintains, supports, and coordinates a national biobanking infrastructure (BBMRI–ERIC, 2016b Art. 4). Being a member of BBMRI–ERIC thereby involves various obligations on various levels that are intended to create a working infrastructure that meets its own aims of harmonizing biobanking practices in Europe and making collections more visible and accessible.

Its ability to improve the visibility and accessibility of sample and data collections is a key promise of BBMRI–ERIC. Various people involved argue that the existence of many smaller biobanks around Europe, which may contain valuable samples and data, is presently unknown. One BBMRI–ERIC staff member lamented that many researchers are not even familiar with biobanks in their own institutions. He explained how he mapped research networks for cancer and bone diseases in Europe onto BBMRI–ERIC membership:

It’s ridiculous […] in the cancer one it’s almost 99 percent, in the bone one it’s a bit less because we miss a few of the countries, otherwise, wherever we have a country, we had also biobanks; so, and they don’t know each other, it came out from the meeting that they literally don’t know that, I don’t know, their, their rare disease ward is in the fourth floor and minus two there is the biobank. (HQ staff member 4)

These problem descriptions point to the diverse forms of visibility BBMRI–ERIC pursues. Not only should researchers know about the distributed availability of samples in Europe, they should also know about biobanks as infrastructures to support their research.

To address the visibility issue, BBMRI–ERIC has sought to build novel relations between biobanks and potential users of biological materials and data. Two software platforms that were largely developed by the German National Node in collaboration with the central IT service should make samples easier to find and access. One is called the ‘Directory’, which is meant to provide an overview of the availability of specific materials in all member biobanks. The other one is called ‘Negotiator’ and is meant to allow researchers who found materials they are interested in via the Directory to negotiate access with the biobank holding these materials. At various occasions, including conference presentations and webinars, BBMRI–ERIC has introduced both platforms to biobanking professionals. Such webinars show how users of the Directory can find specific kinds of samples according to diagnosis (based on ICD codes), the sample type, or certain quality criteria (e.g., accreditations). In one webinar, a representative of BBMRI–ERIC described the Directory as a means of making connections between European biobanks.

However, these webinars also serve as calls to action, in which biobanks are urged to make visible what they have and are willing to share in the Directory. This is because the Directory only shows the information about samples and data that biobanks themselves provide, and only at an aggregate level. Although the Directory is centrally offered and maintained, it depends on individual biobanks to make materials visible. One webinar illustrated the issues this can create by searching for samples with diagnosis C43 (malignant skin melanoma). This resulted in links to 25 biobanks. Yet, the speaker suggested that many biobanks only make data available in broader diagnostic blocs, which he subsequently demonstrated by searching for all diagnoses between C00 and C99 (which covers a range of malignant tissue growth), resulting in links to 98 biobanks (Observation webinar October 2019). BBMRI–ERIC thus largely must rely on the contributions of individual biobanks to realize one of its key tasks—making materials in member institutions visible and accessible. Although the infrastructure’s success in fulfilling this task is contested and difficult to assess, the 2020 Annual Report claims an average of 651 Directory users per month, and 646 total users of the Negotiator—though whether these users would consider their visits successful is not reported.

Common Services IT and ELSI were for a large part developed in the context of the project ADOPT BBMRI–ERIC, which was funded by the European Commission through another infrastructure-specific call (similar to the Preparatory Phase). This project allowed the further development of the infrastructure beyond its formal establishment, serving as an experiment that brought BBMRI–ERIC as an operational research infrastructure into being. One person described ADOPT as an ‘implementation project’ intended to ‘show that we can do biobanking’ (BBMRI staff member 3). On the one hand, one BBMRI–ERIC staff member explained, the project was about ‘watching […] how does it really work. How do you really get the samples and the data out of the biobanks, so we can share again’ (HQ staff member 2). This not only involved the creation of support services such as the IT and ELSI platforms, but also the creation of a cross-national research cohort of 10,000 colorectal cancer cases. BBMRI–ERIC staff member Lukasz Kozera described this effort as follows:

So, we managed to create a huge cohort of 10,000 cases of colorectal cancer, that can be used for any research on this. And I think it’s also very powerful. It shows that the power of BBMRI–ERIC, because you do not access only samples from one country or two countries, but you have let’s say I don’t know like 10-15 countries participating in this. So, that’s really good. I mean the ways of treating and screening patients, it differs between countries. I mean obviously countries like Austria and Belgium, which are really well developed when it comes to medical care, they will provide a different diagnostic or treatment facility comparing for example to Poland or Lithuania…yeah.

This quote illustrates how the project substantiated BBMRI–ERIC’s promise of building large research cohorts, while simultaneously hinting at asymmetries that continue to challenge the idea of a uniform research infrastructure for all of Europe.

Nevertheless, various respondents emphasized the dual benefits ADOPT had in supporting the development of critical infrastructure services and in showing how a distributed European biobanking infrastructure could work. One senior BBMRI–ERIC official stressed how this involved learning various lessons along the way:

We now have a model, if you want to bring together a range of data from twenty countries, what do you confront? Because you have to set up contracts with all these countries and universities, how do you treat the data? It took us two years, but now we know how to do it. And those are very valuable things. (HQ staff member 2)

However, there is a paradox to the importance of the ADOPT project for BBMRI–ERIC. While it is consistent in its claim that ‘it cannot be highlighted often enough that BBMRI–ERIC per se is not a project but a research infrastructure’ (BBMRI–ERIC, 2016a, p. 2), projects appear to be vital in making the infrastructure work.

Aside from the importance of projects in making BBMRI–ERIC operational, there are wider questions about its purpose as a research infrastructure. One question is whether a research infrastructure should primarily do research itself or deliver support services. Although perspectives on this issue differ, many interview respondents shared the impression that BBMRI–ERIC had shifted over the years. Some of them linked this shift to the change from the first Director-General, Jan-Eric Litton, who had been involved in the Preparatory Phase and was in office from 2013 until 2017, to the second, Erik Steinfelder, who entered BBMRI–ERIC from industry and held the position from 2017 until 2020. One former staff member argued that ‘the Assembly [of Members] has, I think, done a reasonably good job in having a more scientific-oriented person at the beginning for setting up everything, and then a more industry-minded person once it went into operation’ (BBMRI staff member 2). Another former staff member, however, saw the shift as less dependent on personnel, and more as a logical development. This person described how ‘from a very young organization, we became mature, and from very scientifically driven, we turned more service-driven’ (HQ staff member 2). Still others date this shift earlier, believing it was the ERIC-status and the more prominent governance role of Member State governments that came with it that moved BBMRI away from its predominantly scientific orientation. All these perspectives suggest that there is a certain logic to research infrastructures shifting from research towards service delivery over time.

This shift in what BBMRI–ERIC promises to do may be reflected in changes in the infrastructure’s communication strategy. During the first few years as an ERIC, descriptions of the infrastructure would state that ‘the ambition of BBMRI–ERIC is to implement a world leading Research Infrastructure for biomedical research in Europe—a true gateway for health’ (*Biobank Europe* 6, p. 18). This idea of a ‘gateway for health’ was also used as the title for the first *Europe Biobank Week* conference in Vienna in 2016. The prominence of *health* in communications was replaced when BBMRI–ERIC re-oriented its public relations strategy in a Vision Paper published in 2018 (BBMRI–ERIC, 2018). It has since presented as its main objective ‘to further build and strengthen value-added sustainable biobanking for all stakeholders enabling academia and industry to make new treatments possible’ (BBMRI–ERIC, 2018c, p. 4). The phrase ‘making new treatments possible’ has since been the cornerstone of the infrastructure’s communication efforts (BBMRI -ERIC, 2018c), appearing prominently in publications (e.g., BBMRI -ERIC, 2019) and on PowerPoint slides used by presenters affiliated with BBMRI–ERIC. The shift from health to *treatments*, as well as more explicitly corporate notions such as ‘value-added’ and key performance indicators (BBMRI–ERIC, n.d.) reflect at least an outward repositioning of what a research infrastructure is expected to be and do.

Not all people involved with BBMRI–ERIC see this as a positive development. Some question the effects it has had on the operations of this distributed European *research infrastructure*. One senior researcher who has long been involved with BBMRI–ERIC, for example, argued that he has seen a lot of positive effects in terms of the presence of biobanks in research policy, but:

If we are very self-critical, I have to say, in our central task, we’re bad. That means, in access to samples and data we only achieved minimal improvements compared to where we started. (BBMRI staff member 3)

By contrast, a BBMRI–ERIC staff member saw the problem with ERICs as currently constituted elsewhere. This person claimed that their active participation in research projects is ‘not sustainable’ and argued that, as research *infrastructures*, they ought to be ‘pillars [that] implement the European Research Area’ (HQ staff member 4) by supplying support services for successful consortia rather than taking part in consortia themselves. This person is in turn contradicted by Georges Dagher, former director of the French national node, who lamented that he ‘did not see a lot of science in BBMRI–ERIC.’ Rather, he believed it ‘paid more attention to integrating more countries in the infrastructure […] we were mimicking the EU.’ In sum, various critics see divergences between the promise of integrating European science and the effects BBMRI–ERIC has had, yet differ in their assessment of what the adequate relation between science and service should have been.

## Conclusion

In this paper I traced how both the organisational contours and narratives around BBMRI–ERIC as a distributed European research infrastructure articulate distinct visions of European scientific integration. Taking this angle on one of the earliest and allegedly largest EU-based ‘research infrastructures’ highlights some of the rhetoric surrounding this form of European research policy. The substance of this notion often remains elusive (Cramer et al., 2020), but the very idea of research infrastructures is highly influential. It is therefore critical to understand what these institutionalised forms of collaboration are claimed to be and how they are (rhetorically) employed in efforts to make Europe through science. To understand these influential entities, I turned to key characteristics of infrastructures discussed in the social sciences, foregrounding the relational, experimental, and promissory character expressed in different interpretations of one European research infrastructure.

European integration and scientific collaboration are jointly constituted in the organisational design of BBMRI–ERIC and its framing as a distributed European research infrastructure. This framing entails various *promises* regarding the potential of integrating European science. The promise of making the many existing biobanks in Europe visible is linked to the strengths attributed to an alleged European tradition in biobanking and initiatives to connect biobanks to each other. In building a single infrastructure out of many European biobanks, BBMRI–ERIC simultaneously created new scientific and European *relations*. Samples and data distributed between different biobanks are (virtually) brought together through forms of European integration that attribute a central role to Member States. These, in turn, engage in new kinds of relations, for example through the infrastructure’s budget. BBMRI–ERIC thus created a scientific Europe characterized by formalized relations between nation-states, biobanks and a central administration. A key question is whether these relations entail a role for BBMRI–ERIC in *doing* or *facilitating* research. This is a highly contentious issue that is closely related to the *experimental* character of European research infrastructure building. The very idea of a research infrastructure for the life sciences was itself considered an experiment, which suggests that the concept of research infrastructures supported by the EU was developed together with its implementation. Throughout this process, the European character of the infrastructure took on a particular shape, in which harmonization efforts paradoxically contributed to a clearer sense of differentiation within the European biobanking landscape. Europe thus continued to be a contested and fragmented category, that continued to be re-defined with the piecemeal, incremental establishment of an infrastructure that relied strongly on the serendipity of, for instance, European funding. While BBMRI–ERIC was imagined and designed to be a force for harmonization in European biobanking, its success in doing so remains unstable and is challenged in the discourses surrounding the infrastructure.

Drawing on different conceptual dimensions of infrastructures gives substance to the category of research infrastructures and their role in efforts to make Europe. Rather than being mere empty policy vessels, the case of BBMRI–ERIC shows that the notion of research infrastructure is open to interpretation and given various meanings. The relation between science and Europe within research infrastructures is shaped by and contested around the promises, relations, and experiments inscribed into them. These include imaginations of European integration through science, which may take on the form of research practice at the bench, or of being competitive in European funding schemes. Similarly, the notion of Europe can take on various forms and shift in meaning even within a single research infrastructure. Although scientific collaboration is sometimes promoted as one of the few uncontroversial remaining success stories of European integration (see e.g., Nature, 2019), the examination of a prominent European research infrastructure presented here suggests otherwise. Key issues for the broader European integration project—such as the relation between intergovernmental and supranational forms of governance, or questions of intra-European differences—surface here in mundane places such as the infrastructure’s statutes, the Partner Charter, the annual budget, and sample search platforms.

Research infrastructures can be considered salient crystallization points for contested discourses of science as an engine of European integration. Often, these contests are disputed in highly specific, technical domains such as budgets or technical platforms, which can be productively explored through the method of ‘infrastructural inversion’ (Bowker & Star, 1999). As European research infrastructures are highly visible in the EU research policy context, they diverge from the often invisible classification systems Bowker and Star discuss. Yet unravelling what research infrastructures entail organisationally, materially and discursively, and attending to controversies over various details of infrastructures can illuminate the complexity of both Europe and science in this domain. Neither Europe nor science are monolithic entities; rather, the question of what is European about science, and what science can do for Europe is continuously (re)imagined, contested and negotiated in the various interpretations and representations of this highly salient European research policy entity.

## Notes

1 My translation. The original quote reads: ‘Stark vertreten sind die skandinavischen Länder, UK und die Niederlande, die dank ihrer Register und Bevölkerungskohorten über sehr gute Ressourcen für die epidemiologische Forschung verfügen.’
